# Rolling Bearing Diagnosis Based on Composite Multiscale Weighted Permutation Entropy

**DOI:** 10.3390/e20110821

**Published:** 2018-10-24

**Authors:** Xiong Gan, Hong Lu, Guangyou Yang, Jing Liu

**Affiliations:** 1 School of Mechanical and Electronic Engineering, Wuhan University of Technology, Wuhan 430070, China; 2 Institute of Agricultural Machinery, Hubei University of Technology, Wuhan 430068, China

**Keywords:** CMWPE, rolling bearing, fault diagnosis

## Abstract

In this paper, composite multiscale weighted permutation entropy (CMWPE) is proposed to evaluate the complexity of nonlinear time series, and the advantage of the CMWPE method is verified through analyzing the simulated signal. Meanwhile, considering the complex nonlinear dynamic characteristics of fault rolling bearing signal, a rolling bearing fault diagnosis approach based on CMWPE, joint mutual information (JMI) feature selection, and k-nearest-neighbor (KNN) classifier (CMWPE-JMI-KNN) is proposed. For CMWPE-JMI-KNN, CMWPE is utilized to extract the fault rolling bearing features, JMI is applied for sensitive features selection, and KNN classifier is employed for identifying different rolling bearing conditions. Finally, the proposed CMWPE-JMI-KNN approach is used to analyze the experimental dataset, the analysis results indicate the proposed approach could effectively identify different fault rolling bearing conditions.

## 1. Introduction

Rolling bearings are one of the most vulnerable parts in mechanical equipment, the working condition of rolling bearing has great influence on the reliability of mechanical system. Therefore, it is valuable to monitor and diagnose the rolling bearing [1].

Recently, various rolling bearing fault diagnosis approaches have been proposed. Non-adaptive time-frequency signal analysis techniques including Wigner–Ville Distribution (WVD) [2], Short-Time Fourier Transformation (STFT) [3], and wavelet-transform (WT) [4], which are widely used to extract feature in fault bearing diagnosis. However, WVD has the problem of cross interference, STFT is merely a single-resolution signal analysis method by using a fixed short-term window function, and WT is difficult to choose suitable wavelet bases. Meanwhile, adaptive time-frequency signal analysis techniques such as Empirical Mode Decomposition (EMD) [5,6], Local Mean Decomposition (LMD) [7,8], and Intrinsic Time-Scale Decomposition (ITD) [9,10] are extensively employed in fault bearing feature extraction. However, these methods generally have the disadvantages of envelope error, mode mixing, and end effect. Furthermore, fault bearing diagnosis method based on image processing [11] has been proposed. Nevertheless, sound data are susceptible to noise from surrounding equipment and environment, the image processing method must convert the vibration data into image data, instead of analyzing the vibration signal directly.

Mechanical systems usually have nonlinear dynamic models due to the instantaneous change of friction, load conditions, friction, and stiffness. For a mechanical condition monitoring system, the vibration signal acquired by the sensor reflects the relevant characteristics of the mechanical system. Different rolling bearing failures cause different mechanical system responses, the vibration signal exhibits complex nonlinear characteristics [12,13]. Lots of nonlinear dynamic approaches such as approximate entropy (APE) [14,15], sample entropy (SE) [16,17], and multiscale sample entropy (MSE) [18,19] have been applied to extract rolling bearing features and identify different rolling bearing conditions.

Bandt et al. [20] first proposed the time series complexity analysis method called permutation entropy (PE), which has higher calculation efficiency and stronger anti-noise ability, compared with APE and SE. PE was adopted for feature extraction of rolling bearing in [21], which indicated that PE could stand for the randomness and dynamic change of vibration signal. In order to evaluate the complexity of time series within different scale factors, Li et al. [22] proposed multiscale permutation entropy (MPE). MPE was also applied to extract fault features and identify different conditions for rolling bearing in [23]. However, PE and MPE ignore the amplitude difference between the same permutation patterns and do not include the amplitude information of the time series. To solve this problem, Fadlallah et al. [24] proposed weight permutation entropy (WPE), which demonstrated better performance in quantifying the information complexity. Yin et al. [25] combined the WPE and multiscale analysis, and proposed the multiscale weighted permutation entropy (MWPE). However, as the scale factor increases, the length of the time series becomes shorter, which results in a sudden change for MWPE [26,27]. In order to improve the statistical reliability, composite multiscale weighted permutation entropy (CMWPE) is put forward in this paper. CMWPE averages the weighted permutation entropy within multiple scale factors, which improves the reliability of entropy estimation. Then, we adopt CMWPE for extracting rolling bearing features. However, not all CMWPE values are closely related to fault information. Joint mutual information (JMI) is applied for selecting the sensitive CMWPE features. In order to intelligently identify different rolling bearing conditions, *k*-nearest-neighbor (KNN) classifier is employed. Therefore, a novel fault diagnosis approach based on CMWPE, JMI, and KNN (simplified into CMWPE-JMI-KNN) is proposed and used to analyze the standard bearing dataset. The analysis results validate the effectiveness of the proposed CMWPE-JMI-KNN method in identifying different fault rolling bearing conditions.

This paper is organized as follows. The MPE, MWPE, and CMWPE are introduced in Section 2, and the superiority of the CMWPE method is also verified in this part by analyzing the simulated signal. In Section 3, the JMI feature selection method is illustrated, and the CMWPE-JMI-KNN method is proposed. In Section 4, experimental validation is presented. In Section 5, conclusions are drawn.

## 2. MPE, MWPE, CMWPE

### 2.1. MPE

#### 2.1.1. PE

Input: Time series X={x(1),x(2),⋯,x(N)}, embedding dimension *m*, time delay *τ*

Output: PE(X,m,τ)

Step 1. For embedding dimension *m*, time delay *τ*, the time series *X* can be reconstructed in phase space as $X^{m,\tau }=\left\{X^{m,\tau }\left(1\right),X^{m,\tau }\left(2\right),\cdots ,X^{m,\tau }\left(k\right),\cdots ,X^{m,\tau }\left(N-\left(m-1\right)\tau \right)\right\}$, $X^{m,\tau }\left(k\right)$ can be expressed as

(1) $$X^{m,\tau }\left(k\right)=\left\{x\left(k\right),x\left(k+\tau \right),\cdots ,x\left(k+\left(m-1\right)\tau \right)\right\}$$

where *k* = 1, 2, …, *N − (m − 1)τ*.

Step 2. Rearrange {x(k),x(k+τ),⋯,x(k+(m−1)τ)} in an increasing order as $\left\{x\left(k+(v_{1}-1)\tau \right)\leq x\left(k+(v_{2}-1)\tau \right)\leq \cdots \leq x\left(k+(v_{m}-1)\tau \right)\right\}$, and obtain the symbol index sequence $\pi_{l}^{m,\tau }=\left[v_{1},v_{2},\cdots ,v_{m}\right]$, $\pi_{l}^{m,\tau }$ is one of the m! distinct symbols ${\left\{\pi_{l}^{m,\tau }\right\}}_{l=1}^{m!}$.

Step 3. Define $p\left(\pi_{l}^{m,\tau }\right)$ as

(2) $$p\left(\pi_{l}^{m,\tau }\right)=\frac{\left\|\left\{k:k\leq N-\left(m-1\right)\tau ,type\left(X^{m,\tau }\left(k\right)=\pi_{l}^{m,\tau }\right)\right\}\right\|}{N-\left(m-1\right)\tau }$$

where type (∙) represents the map from pattern space to symbol space, ‖∙‖ represents the cardinality of a set.

$p\left(\pi_{l}^{m,\tau }\right)$ can be also expressed as

(3) $$p\left(\pi_{l}^{m,\tau }\right)=\frac{\sum_{k=1}^{N-\left(m-1\right)\tau }1_{u:\mathrm{type}\left(u\right)=\pi_{l}^{m,\tau }}\left(X^{m,\tau }\left(k\right)\right)}{\sum_{k=1}^{N-\left(m-1\right)\tau }1_{u:\mathrm{type}\left(u\right)=\Pi }\left(X^{m,\tau }\left(k\right)\right)}$$

where $1_{A}\left(u\right)=\{\begin{array}{cc} 1, & if\;u\;\in \;A\\ 0, & if\;u\notin A \end{array}$, $\Pi ={\left\{\pi_{l}^{m,\tau }\right\}}_{l=1}^{m!}$.

Step 4. PE(X,m,τ) can be expressed as

(4) $$PE\left(X,m,\tau \right)=-\sum_{l:\pi_{l}^{m,\tau }\;\in \;\Pi }p\left(\pi_{l}^{m,\tau }\right)\;\ln\left(p\left(\pi_{l}^{m,\tau }\right)\right)$$

#### 2.1.2. MPE

Input: Time series X={x(1),x(2),⋯,x(N)}, embedding dimension *m*, time delay *τ*, largest scale factor *s_max_*

Output: *MPE*

Initialization: *MPE* = Ø, *s* = 1

Step 1. For X={x(1),x(2),⋯,x(N)}, the coarse-grained time series $y^{s}={\left\{y^{s}\left(j\right)\right\}}_{j=1}^{\left[N/s\right]}$ can be expressed as

(5) $$y^{s}\left(j\right)=\frac{1}{s}\overset{js}{\underset{i=\left(j-1\right)s+1}{\sum }}x\left(i\right)$$

where j=1, 2,⋯,[N/s], [N/s] is the largest positive integer no more than *N/s*.

Step 2. MPE(X,m,τ,s) can be expressed as

(6) $$MPE\left(X,m,\tau ,s\right)=PE\left(y^{s},m,\tau \right)$$

Step 3. MPE=MPE∪MPE(X,m,τ,s), *s* = *s* + 1. Repeat steps 1–3 until *s* is larger than *s_max_*.

### 2.2. MWPE

#### 2.2.1. WPE

Input: Time series X={x(1),x(2),⋯,x(N)}, embedding dimension *m*, time delay *τ*

Output: WPE(X,m,τ)

Step 1. For embedding dimension *m*, time delay *τ*, the time series *X* can be reconstructed in phase space as $X^{m,\tau }=\left\{X^{m,\tau }\left(1\right),X^{m,\tau }\left(2\right),\cdots ,X^{m,\tau }\left(k\right),\cdots ,X^{m,\tau }\left(N-\left(m-1\right)\tau \right)\right\}$, $X^{m,\tau }\left(k\right)$ can be expressed as

(7) $$X^{m,\tau }\left(k\right)=\left\{x\left(k\right),x\left(k+\tau \right),\cdots ,x\left(k+\left(m-1\right)\tau \right)\right\}$$

where *k* = 1, 2, …, *N* − (*m* − 1)*τ*.

Step 2. Rearrange {x(k),x(k+τ),⋯,x(k+(m−1)τ)} in an increasing order as $\left\{x\left(k+(v_{1}-1)\tau \right)\leq x\left(k+(v_{2}-1)\tau \right)\leq \cdots \leq x\left(k+(v_{m}-1)\tau \right)\right\}$, and obtain the symbol index sequence $\pi_{l}^{m,\tau }=\left[v_{1},v_{2},\cdots ,v_{m}\right]$,$\pi_{l}^{m,\tau }$ is one of the m! distinct symbols ${\left\{\pi_{l}^{m,\tau }\right\}}_{l=1}^{m!}$.

Step 3. Define $p_{w}\left(\pi_{l}^{m,\tau }\right)$ as

(8) $$p_{w}\left(\pi_{l}^{m,\tau }\right)=\frac{\sum_{k=1}^{N-\left(m-1\right)\tau }1_{u:\mathrm{type}\left(u\right)=\pi_{l}^{m,\tau }}\left(X^{m,\tau }\left(k\right)\right)w_{k}}{\sum_{k=1}^{N-\left(m-1\right)\tau }1_{u:\mathrm{type}\left(u\right)=\Pi }\left(X^{m,\tau }\left(k\right)\right)w_{k}}$$

where $w_{k}=\frac{1}{m}\overset{m}{\underset{q=1}{\sum }}{\left[x\left(k+\left(q-1\right)\tau \right)-{\bar{X}}^{m,\tau }\left(k\right)\right]}^{2}$, ${\bar{X}}^{m,\tau }\left(k\right)=\frac{1}{m}\overset{m}{\underset{q=1}{\sum }}\left[x\left(k+\left(q-1\right)\tau \right)\right]$.

Step 4. WPE(X,m,τ) can be expressed as

(9) $$WPE\left(X,m,\tau \right)=-\sum_{l:\pi_{l}^{m,\tau }\;\in \;\Pi }p_{w}\left(\pi_{l}^{m,\tau }\right)\;\ln\left(p_{w}\left(\pi_{l}^{m,\tau }\right)\right)$$

#### 2.2.2. MWPE

Input: Time series X={x(1),x(2),⋯,x(N)}, embedding dimension *m*, time delay *τ*, largest scale factor *s_max_*

Output: MWPE

Initialization: *MWPE* = Ø, *s* = 1

Step 1. For X={x(1),x(2),⋯,x(N)}, the coarse-grained time series $y^{s}=\;{\left\{y^{s}\left(j\right)\right\}}_{j=1}^{\left[N/s\right]}$ can be expressed as

(10) $$y^{s}\left(j\right)=\frac{1}{s}\sum_{i=\left(j-1\right)s+1}^{js}x\left(i\right)$$

where j=1, 2,⋯,[N/s].

Step 2. MWPE(X,m,τ,s) can be expressed as

(11) $$MWPE\left(X,m,\tau ,s\right)=WPE\left(y^{s},m,\tau \right)$$

Step 3. MWPE=MWPE∪MWPE(X,m,τ,s), *s* = *s* + 1. Repeat steps 1–3 until *s* is larger than *s_max_*.

### 2.3. CMWPE

Input: Time series X={x(1),x(2),⋯,x(N)}, embedding dimension *m*, time delay *τ*, largest scale factor *s_max_*

Initialization: *CMWPE* = Ø, *s* = 1

Step 1. For X={x(1),x(2),⋯,x(N)}, the coarse-grained time series $y^{s,q}=\;{\left\{y^{s,q}\left(j\right)\right\}}_{j=1}^{\left[\left(N+1\right)/s\right]-1}$ can be expressed as

(12) $$y^{s,q}\left(j\right)=\frac{1}{s}\overset{js+q-1}{\underset{i=\left(j-1\right)s+q}{\sum }}x\left(i\right)$$

where j=1, 2,⋯,[(N+1)/s]−1, q=1, 2,⋯, s.

Step 2. CMWPE averages the WPE values, CMWPE(X,m,τ,s) can be expressed as

(13) $$CMWPE\left(X,m,\tau ,s\right)=\frac{1}{s}\sum_{q=1}^{s}WPE\left(y^{s,q},m,\tau \right)$$

Step 3. CMWPE=CMWPE∪CMWPE(X,m,τ,s), *s* = *s* + 1. Repeat steps 1–3 until *s* is larger than *s_max_*.

**Figure 1:**
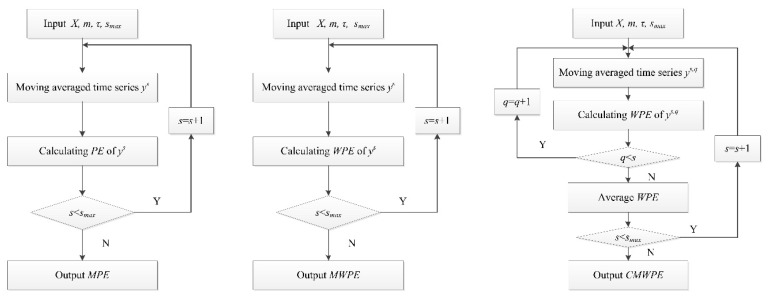
The flowchart of the multiscale permutation entropy (MPE), multiscale weighted permutation entropy (MWPE), and composite multiscale weighted permutation entropy (CMWPE).

Figure 1 shows the flowchart of the MPE, MWPE, and CMWPE.

### 2.4. Comparisons between MPE, MWPE, CMWPE

**Figure 2:**
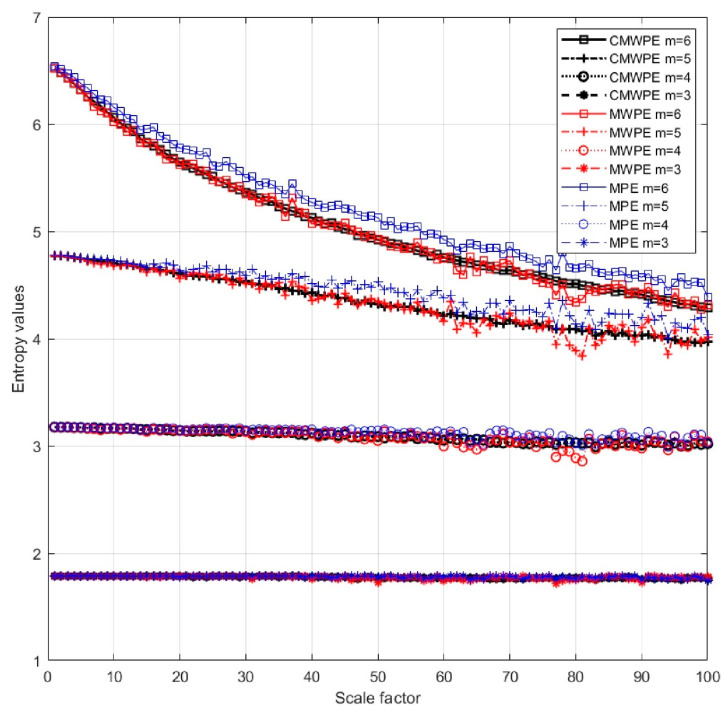
MPE, MWPE, and CMWPE values of the white noise.

To prove the advantage of the CMWPE method, the white noise with 10,000 data points are generated, and we analyze the MPE, MWPE, and CMWPE results on white noise. According to the previous reports [25,28], we select embedding dimension *m* = 3, 4, 5, 6, time delay *τ* = 1, the largest scale factor *s_max_* = 100. The results of MPE, MWPE, and CMWPE on white noise are shown in Figure 2.

As shown, all entropy values increase with the increasing embedding dimension *m*. It is noticeable that, when *m* = 3, 4, with the increase of scale factor, the declines of MPE, MWPE, and CMWPE are not evident, and the superiority of multiscale analysis cannot be displayed effectively. When *m* = 5, 6, with the increasing scale factor, MPE, MWPE and CMWPE all show a significant decreasing trend. In addition, the values of CMWPE and WMPE decrease faster than that of MPE, indicating CMWPE and MWPE can be more sensitive to extract the time series complexity including amplitude information. Furthermore, compared with MWPE, CMWPE can reduce the fluctuation and standard deviation, which demonstrates its superiority. In general, CMWMP not only measures the complexity of time series incorporating amplitude information within multiple scales, but also improves the reliability of entropy evaluation.

## 3. Fault Diagnosis Approach Based on CMWPE, JMI, and KNN

Due to friction, load, impact, and signal transmission interference in mechanical system, not all the CMWPE features are closely related to fault information. If all the CMWPE features are input into the classifier as fault features for identifying bearing condition, the recognition accuracy and efficiency may be decrease. Therefore, sensitive features need to be selected, and CMWPE-JMI-KNN method is proposed in this paper. For CMWPE-JMI-KNN method, JMI algorithm [29,30] is used to select sensitive features, and KNN classifier is applied to identify different fault rolling bearing conditions.

### 3.1. JMI Feature Selection

Give original features set $X=\left\{x_{1},x_{2},\cdots ,x_{s}\right\}$, label information *C*, the size of sensitive features subset *p*, JMI approach is employed to optimize sensitive features subset $Y=\left\{y_{1},y_{2},\cdots ,y_{p}\right\}$. JMI algorithm selects the first sensitive feature *y*_1_ based on the maximum mutual information according to the following expression

(14) $$y_{1}=argmax_{i=1,2,\cdots ,s}\left\{I\left(x_{i};C\right)\right\}$$

where $I\left(x_{i};C\right)$ represents the mutual information of *x_i_* and *C*.

Then, the sequential forward search algorithm is adopted to obtain the sensitive features subset *Y* until the subset size |Y| is equal to *p*. Suppose that *k* features have been selected, choose the *k* + 1 feature $y_{k+1}$ by the following expression

(15) $$y_{k+1}={\mathrm{argmax}}_{x_{i}\;\in \;X-Y}\overset{k}{\underset{j=1}{\sum }}I\left(x_{i};C|y_{j}\right)$$

where $I\left(x_{i};C|y_{j}\right)$ represents the condition mutual information of *x_i_* and *C* under given *y_j_*.

Input: Original features set $X=\left\{x_{1},x_{2},\cdots ,x_{s}\right\}$, label information *C*, the size of sensitive features subset *p*

Output: Sensitive features subset $Y=\left\{y_{1},y_{2},\cdots ,y_{p}\right\}$

Initialization: *Y* = ∅, *k* = 0

Step 1. Select the first sensitive feature $y_{1}$ based on the maximum mutual information, $y_{1}=argmax_{i=1,2,\cdots ,s}\left\{I\left(x_{i};C\right)\right\}$, $Y=\left\{y_{1}\right\}$, *k* = 1.

Step 2. Based on $y_{k+1}={\mathrm{argmax}}_{x_{i}\;\in \;X-Y}\overset{k}{\underset{j=1}{\sum }}I\left(x_{i};C|y_{j}\right)$ to select the *k* + 1 sensitive feature $y_{k+1}$, $Y=Y⋃\left\{y_{k+1}\right\}$, *k* = *k* + 1. Repeat step 2 until the size of sensitive features subset |Y| is equal to *p*.

### 3.2. CMWPE-JMI-KNN

Based on the advantages of CMWPE, JMI, and KNN, the proposed CMWPE-JMI-KNN approach can be described as follows.

Input: Training samples, training label *L_train_*, testing samples, embedding dimension *m*, time delay *τ*, maximum scale factor *s_max_*, the number of sensitive features *p*

Output: Testing label *L_test_*

Step 1. Calculating CMWPE of the training samples to form training matrix *T_train_*, adopting JMI method to select the first *p* features to construct into sensitive low-dimension matrix *T_train_*_,*JMI*_.

Step 2. Calculating CMWPE of the testing samples to form testing matrix *T_test_*, forming the sensitive low-dimension matrix *T_test_*_,*JMI*_ based on the selected results in training samples.

Step 3. Input *T_train_*_,*JMI*_, *T_test_*_,*JMI*_, *L_train_* to the KNN classifier for identifying the rolling bearing conditions of testing samples, output the testing label *L_test_*.

**Figure 3:**
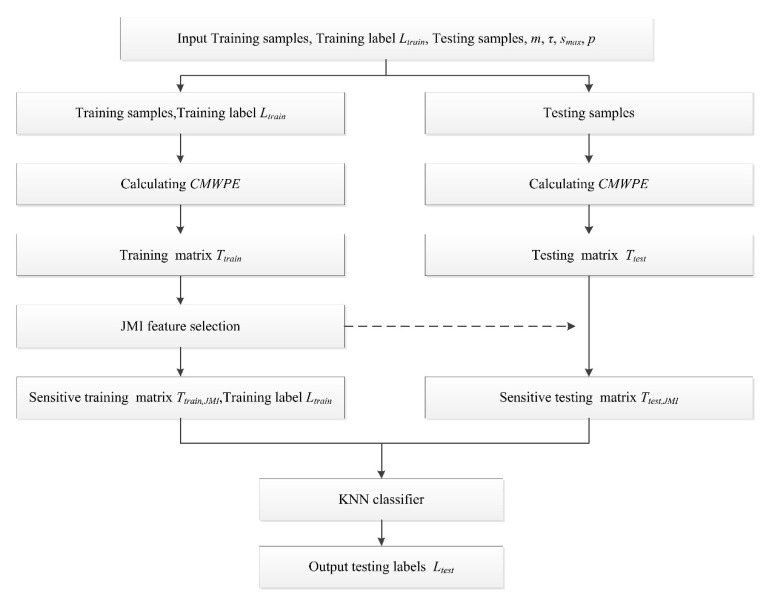
Flowchart of the proposed CMWPE-JMI-KNN method. JMI, joint mutual information; KNN, *k*-nearest neighbor.

Figure 3 shows the flowchart of the proposed CMWPE-JMI-KNN method.

## 4. Experimental Validation

**Figure 4:**
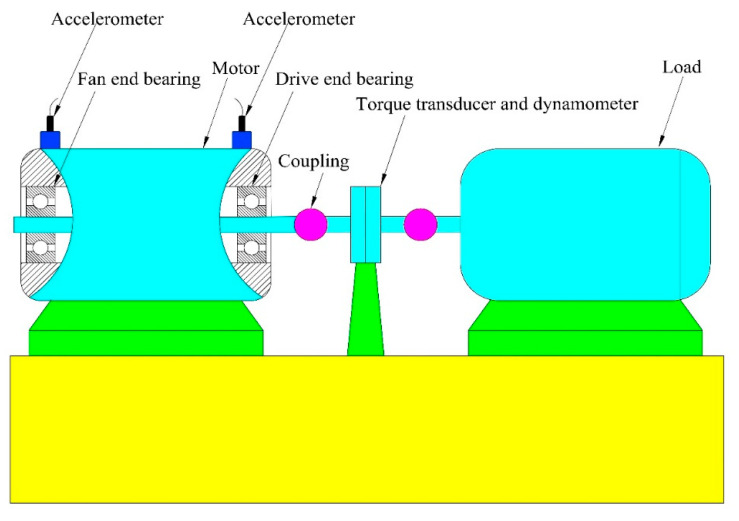
Schematic diagram of bearing test bench.

Standard experiment dataset is provided by Rolling Bearing Data Center of Case Western Reserve University [31]. The proposed CMWPE-JMI-KNN method is applied to analyze the dataset for verifying its effectiveness. Figure 4 shows the schematic diagram of bearing test bench, the experiment uses 6205-2RS JEM SKF bearing and adopts accelerometer to collect the vibration signal.

**Figure 5:**
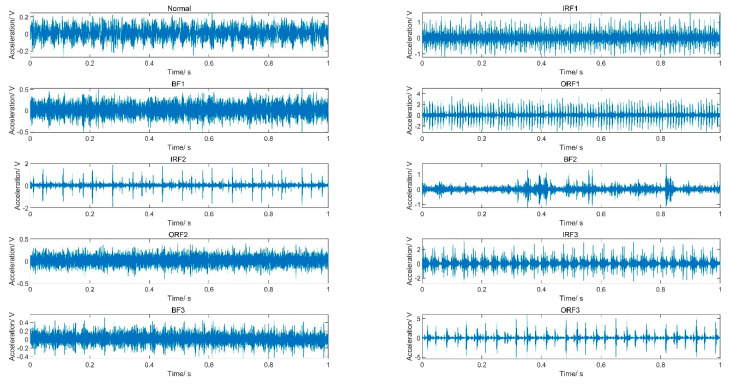
Vibration data under 10 conditions.

The standard dataset contains vibration data of the drive end bearing under ten conditions, including normal condition (noted into Normal), inner race fault conditions with fault size 0.1778 mm, 0.3556 mm, 0.5334 mm (noted into IRF1, IRF2, IRF3), ball fault conditions with fault size 0.1778 mm, 0.3556 mm, 0.5334 mm (noted into BF1, BF2, BF3), outer race fault conditions with fault size 0.1778 mm, 0.3556 mm, 0.5334 mm (noted into ORF1, ORF2, ORF3). Figure 5 shows the vibration data under ten conditions. The sampling frequency is 12 kHz, the motor speed is 1772 rpm, and the load is 0 HP. Due to friction, load, shock, and noise interference, it is hard to identify concrete bearing condition from the time domain waveform.

The vibration data are divided into multiple data samples without overlap. Each condition has 110 samples, and every sample contains *N* = 1024 points. Twenty-two randomly selected samples in each condition were used to form the training samples, and the remaining 88 samples were used as testing samples. The detailed information of the training samples and testing samples are shown in Table 1. Here, the embedding dimensions *m* = 3, 4, 5, 6, time delay *τ* = 1, and maximum scale factor *s_max_* = 100, sensitive features size *p* = 10. The proposed CMWPE-JMI-KNN method is applied to analysis the data, which can be described as follows.

Step 1. Calculate CMWPE of the training samples to form training matrix *T_train_* ∈ R^22×1000^. Sort 100 features using JMI, and select the first 10 features to construct into sensitive low-dimension matrix *T_train_*_,*JMI*_ ∈ R^22×100^.

Step 2. Calculate CMWPE of the testing samples to form testing matrix *T_test_* ∈ R^88×1000^, and obtain the sensitive low-dimension matrix *T_test_*_,*JMI*_ ∈ R^88×100^ based on the selected results in the training samples.

Step 3. Input *T_train_*_,*JMI*_, *T_test_*_,*JMI*_ and *L_trian_* to the KNN classifier (*k* = 1) for identifying different conditions of the testing samples, output the testing label *L_test_*.

**Figure 6:**
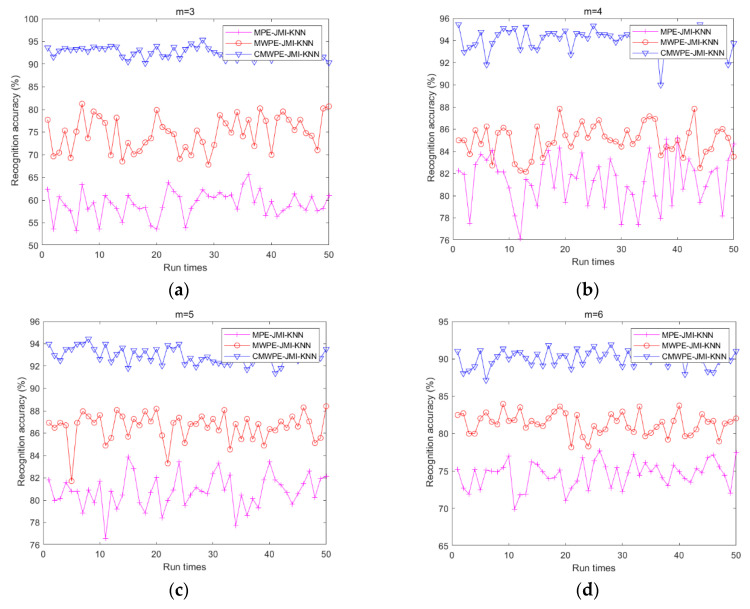
Recognition accuracy of the three methods. (**a**) *m* = 3; (**b**) *m* = 4; (**c**) *m* = 5; (**d**) *m* = 6.

To prove the effectiveness of the proposed CMWPE-JMI-KNN method and the advantage of CMWPE, CMWPE is substituted with MPE and MWPE in CMWPE-JMI-KNN to extract the fault bearing features. Similarly, JMI is employed to select sensitive features and KNN classifier is applied for recognizing the different bearing conditions. Under the same experimental settings, all the three methods were conducted with 50 run times for comparison. The recognition results are shown in Figure 6 and Table 2. As demonstrated, for *m* = 3, 4, 5, 6, CMWPE-JMI-KNN is superior to MWPE-JMI-KNN and MPE-JMI-KNN in recognition ability. Especially, the maximum recognition accuracy of CMWPE-JMI-KNN reaches to 95.45% with *m* = 4. In addition, the recognition accuracy of MWPE-JMI-KNN method is less than that of CMWPE-JMI-KNN, which obtains the moderate recognition accuracy. Furthermore, the MPE-JMI-KNN method has the worst recognition accuracy. The results validate the effectiveness of the proposed CMWPE-JMI-KNN method in recognizing different fault conditions, and the advantage of CMWPE in extracting fault bearing features.

**Figure 7:**
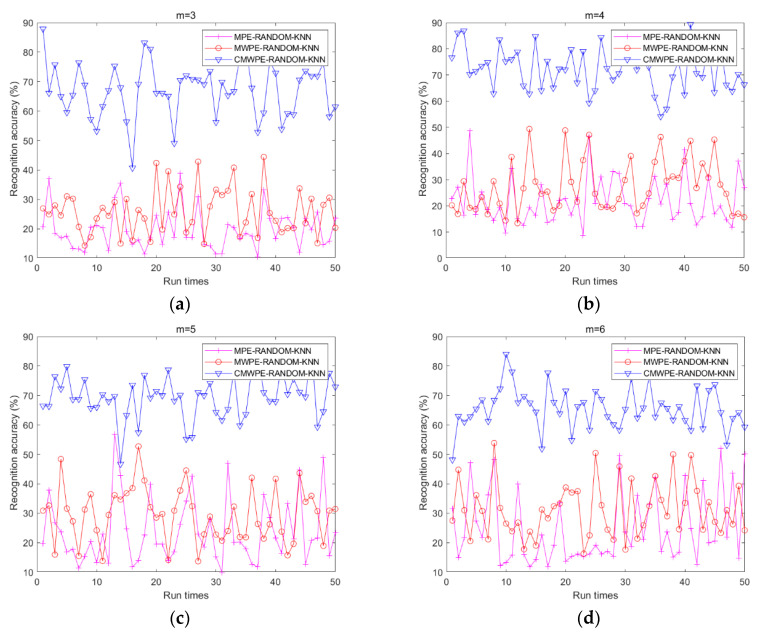
Recognition accuracy of the three methods with random selected features. (**a**) *m* = 3; (**b**) *m* = 4; (**c**) *m* = 5; (**d**) *m* = 6.

To further display the necessity of JMI feature selection, random feature selection method is utilized to replace JMI in CMWPE-JMI-KNN, MWPE-JMI-KNN, and MPE-JMI-KNN methods to randomly select 10 features. Similarly, CMWPE, MWPE, and MPE are used for extracting the bearing features, and KNN classifier is employed to recognize the different bearing conditions. For comparison, all the three methods combined with random selected features were conducted with 50 run times under the same experimental settings. The recognition results are shown in Figure 7 and Table 3. It was found that all the recognition accuracies of three methods with random feature selection are lower than those of integrated with JMI, which reveals the necessity of JMI in selecting sensitive features. Furthermore, among these three methods, CMWPE-RANDOM-KNN method achieved the highest recognition accuracy with *m* = 3, 4, 5, 6, which further demonstrates the advantage of the CMWPE for feature extraction.

**Figure 8:**
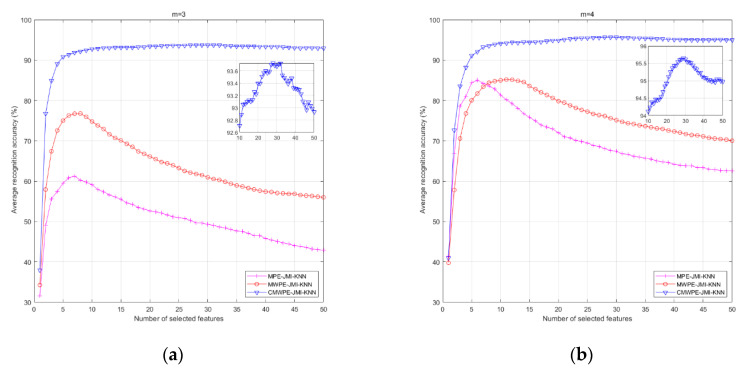

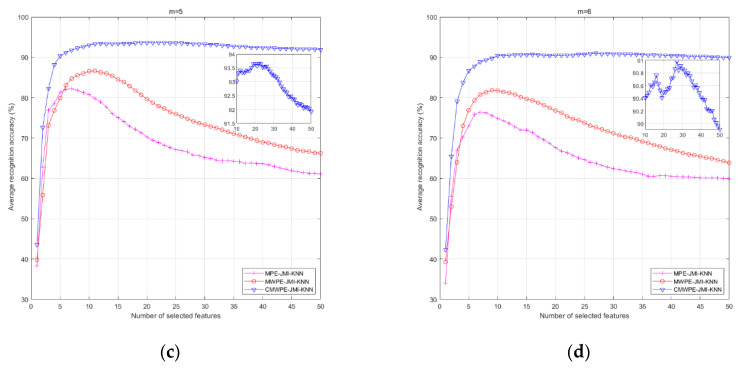
The average recognition accuracy with various number of selected features. (**a**) *m* = 3; (**b**) *m* = 4; (**c**) *m* = 5; (**d**) *m* = 6.

In order to analyze the relationship between the number of selected features and recognition accuracy, all the three methods were conducted with 50 run times to obtain average recognition accuracy for each number of selected features. Figure 8 shows the corresponding average recognition accuracy for various numbers of selected features with *m* = 3, 4, 5, 6. It was found that, compared with MWPE-JMI-KNN and MPE-JMI-KNN, the CMWPE-JMI-KNN method achieved much higher recognition accuracy. Especially when *m* = 4, the proposed CMWPE-JMI-KNN method achieved the highest recognition accuracy as 95.66% when the first 29 sensitive features were selected by JMI. The analysis results verify the advantage of the proposed CMWPE-JMI-KNN method. In addition, too large or too small selected features will lead to the decline in the recognition accuracy. For the CMWPE-JMI-KNN method, when *m* = 3, 4, 5, 6, the optimal number of sensitive features are 28, 29, 22, and 27, respectively. The reason is that, it contains less fault information if too small selected sensitive features are selected. On the contrary, if the number of selected sensitive features is too large, it will lead to redundancy of fault information and reduce the recognition accuracy.

**Figure 9:**
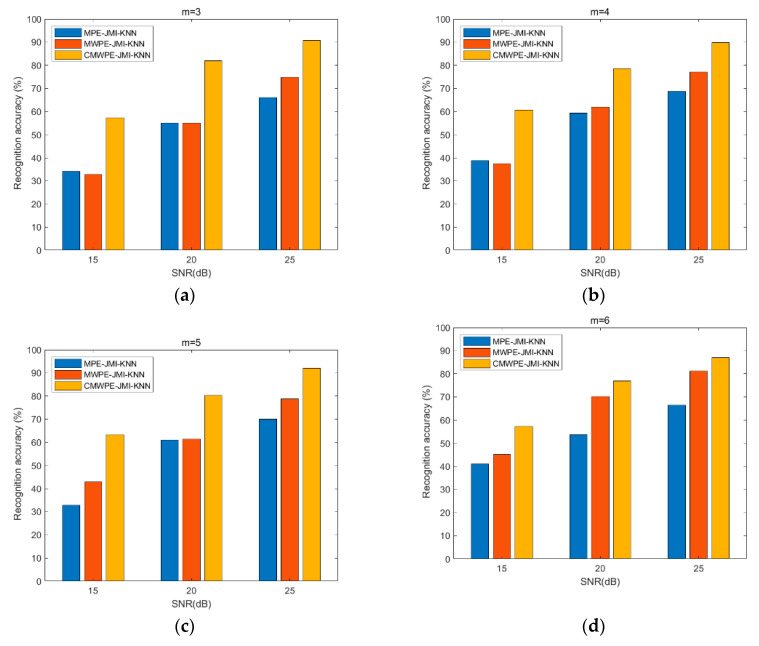
Recognition accuracy of the three methods under different SNR (signal-to-noise ratio) conditions. (**a**) *m* = 3; (**b**) *m* = 4; (**c**) *m* = 5; (**d**) *m* = 6.

To test the noise robustness of the CMWPE-JMI-KNN method, random noise is added to the vibration signal of the rolling bearing. Figure 9 shows the identification results for the CMWPE-JMI-KNN, MWPE-JMI-KNN, and MPE-JMI-KNN methods under different SNR (signal-to-noise ratio) conditions. It can be seen from Figure 9 that under the same SNR condition, the fault recognition performance of the CMWPE-JMI-KNN method is better than the other two fault diagnosis methods. Moreover, the larger the SNR, the higher the identification accuracy for the three methods. Especially, when SNR = 25 dB, the proposed CMWPE-JMI-KNN method achieves the highest recognition accuracy as 90.68% with *m* = 3.

## 5. Conclusions

In this paper, a novel nonlinear dynamic approach named CMWPE is put forward, the comparison results verify the superiority of CMWPE method by analyzing the simulated signal. Based on the virtues of CMWPE, JMI, and KNN, the CMWPE-JMI-KNN approach for recognizing the fault bearing conditions is presented and employed to experimental dataset analysis. The analysis results validate the effectiveness of the proposed CMWPE-JMI-KNN approach, the advantage of CMWPE for extracting fault bearing information, and the necessity of JMI feature selection. The subsequent research will be focused on as follows:

1. Combining entropy theories and advanced signal processing techniques to further improve the recognition accuracy and anti-noise ability.
2. Applying the proposed diagnosis method to more types of mechanical fault diagnosis in real world industrial applications.

## Figures and Tables

**Table 1:** The detailed information of the training samples and testing samples.

Bearing Condition	Fault Diameter (mm)	Motor Load (HP)	Motor Speed (rpm)	Label	Number of Training Samples	Number of Testing Samples
Normal	0	0	1772	1	22	88
IRF1	0.1778	0	1772	2	22	88
BF1	0.1778	0	1772	3	22	88
ORF1	0.1778	0	1772	4	22	88
IRF2	0.3556	0	1772	5	22	88
BF2	0.3556	0	1772	6	22	88
ORF2	0.3556	0	1772	7	22	88
IRF3	0.5334	0	1772	8	22	88
BF3	0.5334	0	1772	9	22	88
ORF3	0.5334	0	1772	10	22	88

**Table 2:** Recognition accuracy of the three methods.

Experiments	CMWPE-JMI-KNN			MWPE-JMI-KNN			MPE-JMI-KNN		
	Accuracy (%)			Accuracy (%)			Accuracy (%)		
	Max	Min	Mean	Max	Min	Mean	Max	Min	Mean
*m* = 3	95.34	90.22	92.70	81.25	67.84	74.79	65.68	53.18	59.13
*m* = 4	95.45	90.00	94.11	87.84	82.15	85.00	85.22	76.13	81.35
*m* = 5	94.43	91.36	93.01	88.40	81.70	86.56	83.86	76.59	80.79
*m* = 6	93.06	87.15	90.17	83.97	78.18	81.38	77.72	69.88	74.55

**Table 3:** Recognition accuracy of the three methods with random selected features.

Experiments	CMWPE-RANDOM-KNN			MWPE-RANDOM-KNN			MPE-RANDOM-KNN		
	Accuracy (%)			Accuracy (%)			Accuracy (%)		
	Max	Min	Mean	Max	Min	Mean	Max	Min	Mean
*m* = 3	87.95	40.68	67.10	44.43	14.31	25.88	38.97	10.45	19.74
*m* = 4	89.43	54.20	72.18	49.43	13.40	27.48	48.63	8.63	22.02
*m* = 5	86.47	46.70	69.56	52.72	13.75	29.57	56.93	10.00	23.91
*m* = 6	84.09	48.18	65.48	53.86	16.36	31.15	52.15	11.81	24.94

## References

[1] Zheng J., Cheng J., Yang Y. (2013). A rolling bearing fault diagnosis approach based on LCD and fuzzy entropy. Mech. Mach. Theory.

[2] Gong H., Huang W., Zhao K., Li S., Zhu Z. (2011). Time-frequency feature extraction based on fusion of Wigner-Ville distribution and wavelet scalogram. J. Vib. Eng..

[3] Gao H., Liang L., Chen X., Xu G. (2015). Feature extraction and recognition for rolling element bearing fault utilizing short-time Fourier transform and non-negative matrix factorization. Chin. J. Mech. Eng..

[4] Rodriguez N., Cabrera G., Lagos C., Cabrera E. (2017). Stationary Wavelet Singular Entropy and Kernel Extreme Learning for Bearing Multi-Fault Diagnosis. Entropy.

[5] Lei Y., Lin J., He Z., Zuo M.J. (2013). A review on empirical mode decomposition in fault diagnosis of rotating machinery. Mech. Syst. Signal Process..

[6] Li Y., Xu M., Wei Y., Huang W. (2015). An improvement EMD method based on the optimized rational Hermite interpolation approach and its application to gear fault diagnosis. Measurement.

[7] Zhang Y., Qin Y., Xing Z., Jia L., Cheng X. (2013). Roller bearing safety region estimation and state identification based on LMD–PCA–LSSVM. Measurement.

[8] Liu H., Han M. (2014). A fault diagnosis method based on local mean decomposition and multi-scale entropy for roller bearings. Mech. Mach. Theory.

[9] Zhang L., Li P., Li M., Zhang S., Zhang Z. (2014). Fault diagnosis of rolling bearing based on ITD fuzzy entropy and GG clustering. Chin. J. Sci. Instrum..

[10] Yang Y., Pan H., Ma L., Cheng J. (2014). A roller bearing fault diagnosis method based on the improved ITD and RRVPMCD. Measurement.

[11] Lu C., Wang Y., Ragulskis M., Cheng Y. (2016). Fault Diagnosis for Rotating Machinery: A Method based on Image Processing. PLoS ONE.

[12] Zhao L.Y., Wang L., Yan R.Q. (2015). Rolling Bearing Fault Diagnosis Based on Wavelet Packet Decomposition and Multi-Scale Permutation Entropy. Entropy.

[13] He W., Zi Y., Chen B., Wu F., He Z. (2015). Automatic fault feature extraction of mechanical anomaly on induction motor bearing using ensemble super-wavelet transform. Mech. Syst. Signal Process..

[14] Pincus S. (1995). Approximate entropy (ApEn) as a complexity measure. Chaos.

[15] Li Q., Wang T.Y., Xu Y.G., He H.L., Zhang Y. (2007). Fault diagnosis of rolling bearings based on chaos and two-dimensional approximate Entropy. J. Vib. Eng..

[16] Richman J.S., Moorman J.R. (2000). Physiological time-series analysis using approximate entropy and sample entropy. Am. J. Physiol.-Heart C.

[17] Zhao Z.H., Yang S.P. (2012). Sample entropy-based roller bearing fault diagnosis method. J. Vib. Shock.

[18] Costa M., Goldberger A.L., Peng C.K. (2007). Multiscale Entropy Analysis of Complex Physiologic Time Series. Phys. Rev. Lett..

[19] Jinde Z., Junsheng C., Yang Y. (2012). A rolling bearing fault diagnosis approach based on multiscale entropy. J. Hum. Univ..

[20] Bandt C., Pompe B. (2002). Permutation Entropy: A Natural Complexity Measure for Time Series. Phys. Rev. Lett..

[21] Rao G.Q., Feng F.Z., Si A.W., Xie J.L. (2014). Method for optimal determination of parameters in permutation entropy algorithm. J. Vib. Shock.

[22] Duan L., Xiaoli L., Zhenhu L., Logan J.V., Jamie W.S. (2010). Multiscale permutation entropy analysis of EEG recordings during sevoflurane anesthesia. J. Neural Eng..

[23] Li Y., Xu M., Wei Y., Huang W. (2016). A new rolling bearing fault diagnosis method based on multiscale permutation entropy and improved support vector machine based binary tree. Measurement.

[24] Fadlallah B., Chen B., Keil A., Príncipe J. (2013). Weighted-permutation entropy: A complexity measure for time series incorporating amplitude information. Phys. Rev. E.

[25] Yin Y., Shang P. (2014). Weighted multiscale permutation entropy of financial time series. Nonlinear Dyn..

[26] Wu S.D., Wu C.W., Lin S.G., Wang C.C., Lee K.Y. (2013). Time Series Analysis Using Composite Multiscale Entropy. Entropy.

[27] Zheng J., Pan H., Cheng J. (2017). Rolling bearing fault detection and diagnosis based on composite multiscale fuzzy entropy and ensemble support vector machines. Mech. Syst. Signal Process..

[28] Yin Y., Shang P. (2017). Multivariate weighted multiscale permutation entropy for complex time series. Nonlinear Dyn..

[29] Yang H., Moody J. (2000). Data Visualization and Feature Selection: New Algorithms for Nongaussian Data. Adv. Neural Inf. Process. Syst..

[30] Sheng S., Yang H., Wang Y., Pan Y., Tang J. (2015). Joint mutual information feature selection for underwater acoustic targets. J. Northwest. Polytech. Univ..

[31] Bearing Data Center, Case Western Reserve University. http://csegroups.case.edu/bearingdatacenter/pages/download-data-file.

